# Biohybrid microcapsules based on electrosprayed CS-immobilized nanoZrV for self-healing epoxy coating development

**DOI:** 10.1039/d4ra02289k

**Published:** 2024-06-10

**Authors:** Lydia Uko, Marwa Elkady

**Affiliations:** a Chemical and Petrochemicals Engineering Department, Egypt-Japan University of Science and Technology Alexandria 21934 Egypt lydia.ukochile@ejust.edu.eg; b Fabrication Technology Department, Advanced Technology and New Materials Research Institute (ATNMRI), City of Scientific Research and Technology Applications Alexandria 21934 Egypt

## Abstract

In this work, zirconium vanadate nanoparticles were immobilized into chitosan using a facile electrospraying technique to produce CS–ZrV hybrid microcapsules for the development of a self-healing coating. Upon assessment, hybrid microcapsules possessed desirable properties with a mean particle size of 319 μm, maintaining good thermal stability of ∼55% at 700 °C, and were subsequently incorporated into an epoxy resin to develop a biocompatible self-healing coating, CZVEx, for carbon steel corrosion protection. Scratched samples of self-healing and control coatings were analyzed in a corrosion medium of 3.5 wt% aqueous NaCl. SEM images of the scratched coating sample, after days of immersion, revealed healing of defects through the appearance of an epoxide gel-like substance due to the release of polymeric vanadate that reacted with corrosion agents, resulting in polymerization of vanadium hydrates and subsequent self-healing, validated by the proposed mechanism of self-healing. Electrochemical impedance spectroscopy analysis further confirmed CZVEx coating possessed excellent self-healing capabilities through a significant impedance rise from 4.48 × 10^5^ to 5.52 × 10^5^ (ohm cm^2^) between the 7th and 14th day of immersion. Furthermore, comparative polarization assessment of coating samples with/without defects indicated the accuracy of EIS for self-healing analysis, and showed the sample with no defect was only 2.6 times more corrosion resistant than the scratched coating, as against bare steel substrate that was 22 times less resistant, revealing superior self-healing anticorrosion properties of the coating.

## Abstract 2

In this work, zirconium vanadate nanoparticles were immobilized into chitosan using a facile electrospraying technique to produce CS–ZrV hybrid microcapsules for the development of a self-healing coating.

## Graphical abstract

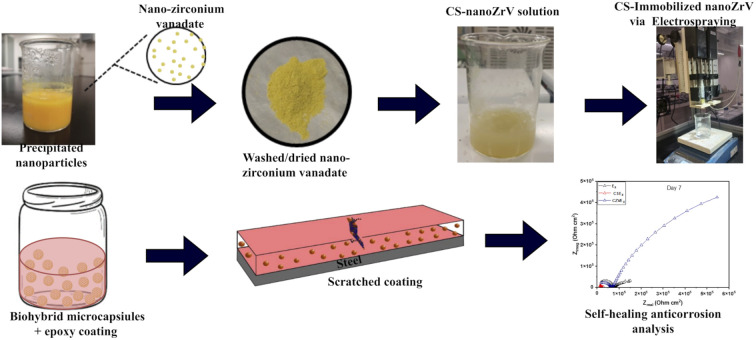

## 1 Introduction

Carbon steel, owing to its desirable properties, is largely used as one of the structural materials in building and construction across the globe. However, wide commercial application of carbon steel is limited due to severe corrosion attacks, which cause substantial economic loss and safety issues. Several strategies have been employed to protect carbon steel, including cathodic protection,^1^ corrosion inhibitors,^2^ and protective surface coating.^3,4^ Amongst these strategies, the most economical and common for the corrosion protection of the metal is the conventional protective surface coating, which induces a barrier between the metals and their immediate environment. Nevertheless, the protective ability of the coating is lost once there is damage arising from mechanical effects like abrasions or sudden impact. The proposal of a novel solution, a self-healing mechanism, is regarded as the best way to fortify conventional coatings to be smarter in protecting metallic structures and prolonging their service life. The most practical way stipulated to achieve self-healing is through the incorporation of micro/nanocapsules filled with healing agents into the barrier coatings to release the healing agents on the occurrence of damage.^5,6^

Micro/nanocapsules containing healing agents have been extensively employed in polymeric coatings for the protection of metals, and are known to provide only a one-time healing effect. Since, on propagation of the defect, the capsules get broken and release the agent, which polymerizes with the coating triggering healing of the defect. In recent times, a successful alternative proposed is the use of efficient organic coating matrices modified by metal oxide inhibitors^7^ or nanoparticles.^8,9^ It has been recognized that the use of inorganic metal oxides is the easiest technique for preventing corrosion.^10^ The reason is that metal oxides have superior adherence to metal substrates, and were found to possess the ability to generate a huge number of van der Waals interactions that induce stable covalent bonds.^11^

Epoxy, a common protective organic coating, possesses higher bond strength formed within its structure that makes it desirable for use as protective coatings. The chemical structure of polymeric materials depicting linkages and bonding between its structural regions plays a key role in developing polymers with self-healing capabilities. Thus, when metal oxides are added to polymeric materials, the number of hydrogen bonding and intermolecular interactions increase, inducing a self-healing effect.^12^ Epoxy resins are thermosetting polymers that possess superior mechanical and adhesive properties that have widely encouraged their use as protective coatings and as matrices in structural composite materials. These excellent properties do not prevent their vulnerability to damage in the form of microcracks due to their brittleness.^13^ Several efforts have been made to improve the coatings’ fracture toughness *via* the incorporation of nano/microscale metal oxide (CeO_2_ and ZrO_2_) particles,^14^ which is not sufficient as there is no way to halt the ingression of corrosion agents on eventual propagation of microcrack that is inevitable due to handling and environmental conditions. Subsequently, metal oxide inorganic particles are modified with organic materials to impart self-healing capabilities to them. Yurkevich *et al.*^15^ stated that self-healing could be realized with inorganic materials by loading inorganic nanoparticles into organic materials, such that upon a defect in the system, harmonious chemical and physical effects will drive interactions and eventually close the defect. Such organic–inorganic coating systems have been found to significantly improve the corrosion protection of developed coatings while providing autonomous healing. For instance, Ghomi *et al.*^16^ developed self-healing epoxy coating by dispersing titanium oxide (TiO_2_) nanoparticles into mixed acrylamide and propyl methacrylate polymers to obtain composite nanogels that were incorporated into epoxy coating. They observed that the presence of the nanogel composite enhanced the anti-corrosion and self-healing performance of the epoxy coating.

Given the problematic effects such as toxicity and carcinogenicity of some polymeric materials for loading corrosion inhibitors or healing agents, and the need for eco-friendly technologies, biocompatible and biodegradable polymer matrices like chitosan and others have been explored for the production of composite materials employed in self-healing coatings.^17^ Chitosan (CS) is a natural biopolymer that is non-toxic, biocompatible, biodegradable, possesses antimicrobial and good film-forming abilities, and has been employed as a functional supporting matrix for composite formulations of different shapes and designs, and for different applications including self-healing anticorrosion.^17,18^

The use of zirconium vanadate (ZrV_2_O_7_, *i.e.*, ZrV) nanoparticles in polymer coating matrix or for fabrication of organic–inorganic material in coating systems for corrosion protection has been sparsely reported. The closest studies are on the use of ZrV as cathode electrode material in Li-ion batteries and supercapacitors.^19^ However, organic–inorganic hybrid materials with ZrV as the inorganic part have been reported for ion exchanger fabrication.^20,21^ Meaning, ZrV are largely employed as ion exchangers. All the same, vanadate (V_2_O_5_) has been widely reported as an effective corrosion inhibitor^22,23^ and for conversion coatings,^24,25^ and was recently reported for use in the fabrication of hybrid V_2_O_5_@polyaniline–tannic acid nanocomposite, which was incorporated into waterborne epoxy for corrosion protection, and was reported to provide significant anticorrosion and self-healing performance.^26^ Also, CS–vanadate composite systems have been reported for some applications.^27,28^ However, no study has yet reported the use of ZrV composited with CS as an organic–inorganic composite for the development of self-healing and anticorrosion epoxy coating. Also, the fabrication of CS–ZrV hybrid microcapsules *via* electrospraying technique is yet to be reported. Hence, the use of CS–ZrV hybrid microcapsules for self-healing anticorrosion coating applications is hereby reported.

In the present study, we report on the fabrication of hybrid (CS–ZrV) microcapsules for self-healing and anticorrosion coating development. The composite microcapsules were prepared *via* a simple electrospraying technique employing the crosslinking action of tripolyphosphate (TPP). Successful preparation of composite materials was assessed using XRD, EDX, FTIR, and TGA, and subsequently incorporated into epoxy coating matrix to develop self-healing anticorrosion coating. Coatings were analyzed electrochemically to determine their corrosion protection capabilities.

## 2 Materials and methods

### 2.1 Materials

Zirconium oxychloride octahydrate (ZrOCl_2_·8H_2_O) and urea were obtained from Dop Organik Kimya San Ve Tic Ltd, Chitosan (CS) (100–300 kDa molecular weight) was purchased from ACROS Organics, USA. Sodium metavanadate (NaVO_3_), ≥99% glacial acetic, ammonium hydroxide, hydrochloric acid, and analytical acetone were bought from Sigma-Aldrich, Germany. Sodium tripolyphosphate (Alpha Chemika, India), solvent-free transparent epoxy resin (Kemapoxy 150 A) and its hardener (CMB group, Egypt), and sodium chloride (CDH Ltd, New Delhi, India) are other materials used.

### 2.2 Preparation of ZrV nanoparticles

**Fig. 1:**
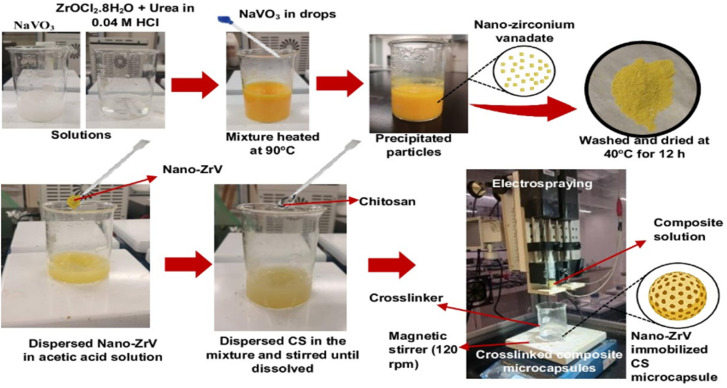
Preparation scheme of ZrV nanoparticle and CS–ZrV composite microcapsules *via* electrospraying method.

The precipitation method reported in the literature^29^ was followed. Briefly, a stock solution of sodium metavanadate (NaVO_3_) was first prepared by dispersing 4.877 g in 100 ml of distilled water and stirring until fully dissolved. Also, a stock solution of zirconium oxychloride (ZrOCl_2_·8H_2_O) was prepared by dissolving 3.221 g in 100 ml of 0.04 M HCl solution and stirred (Fig. 1); 1.5 g of urea was added to this mixture under continuous stirring until a homogenous solution was obtained. Then, NaVO_3_ solution was slowly added to the ZrOCl_2_ mixture in drops leading to the formation of the precipitate. The precipitate solution was heated to 90 °C and left at this temperature for 1 h, it was further centrifuged to separate the precipitate from the liquid phase. Collected ZrV nanoparticles were finally washed with distilled water severally to remove any residual ions. After which it was dried at 40 °C for 24 h.

### 2.3 Preparation of CS–ZrV composite microcapsules

Employing the electrospraying technique, described in a previous study,^30^ CS–ZrV microcapsules were prepared thus (Fig. 1): 0.4 g of ZrV nanoparticles was dispersed in 100 ml solution of 1.2% v/v acetic acid and stirred vigorously for 30 min. Subsequently, CS, 4 g, was added to the mixture and stirred until CS was completely dissolved. The mixture was left to stir at 200 rpm for 12 h until degassed to ensure the nanoparticles completely homogenize with CS and stall their aggregation within the mixture. Tripolyphosphate was dissolved separately to obtain a 3 wt% solution for cross-linking. The composite viscous solution was finally fed into a syringe with an attached 27 G needle and placed into the digital pump of the electrospray device and sprayed at electrospray parameters of 18.7 kV, 7 ml h^−1^ flow rate, and 8 cm TTC distance into the cross-linker contained in a beaker and placed on a magnetic stirrer, which was operating at 120 rpm. Afterward, the formed CS–ZrV microcapsules composite was left to crosslink for about 12 h before washing with distilled water and dried at 27 °C overnight in a dryer (Taisite Lab Sciences Inc).

### 2.4 Characterization of ZrV nanoparticles and CS–ZrV composite microcapsules

To determine the chemical structure of the synthesized nanoparticles and the hybrid microcapsules, FTIR spectrophotometer (Brucker Scientific Instruments, Vertex 70, Germany) was used to obtain the spectra of the samples. Malvern diffractometer (Malvern Panalytical Ltd, Malvern, UK) was used to obtain the XRD diffractogram of the samples. The physical properties and micromorphological features of the microcapsules were determined using an energy-dispersive X-ray spectrometer (EDX) and a scanning electron microscope (SEM) (JCM-6000Plus, JEOL Electronics Co. Ltd, Tokyo, Japan), respectively. The mean size of the microcapsules was assessed from the SEM images using Image-J software. JEOL's JEM-2100F, Japan, was used to carry out the transmission electron microscopy (TEM). A thermal analyzer (STA PT1600, LINSEIS, Germany) was utilized to assess the thermal stability of the microcapsules *via* thermogravimetric analysis (TGA) under an argon atmosphere at a heating rate of 10 °C min^−1^ and a temperature range of 25 to 700 °C.

### 2.5 Preparation of self-healing coating

**Fig. 2:**
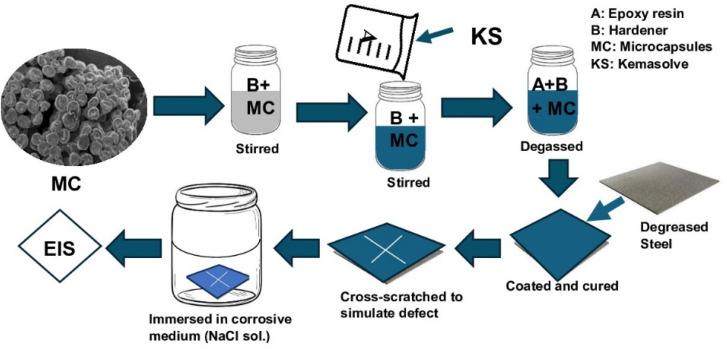
Preparation of the self-healing coating.

The self-healing coating was prepared (Fig. 2) by adding the microcapsules to the epoxy hardener and stirring for 30 min at 200 rpm, after which epoxy resin was added and stirred for another 10 min. Epoxy resin was diluted with 5% kemasolvent relative to the resin's quantity to reduce viscosity before use. The quantity of microcapsule was 1 g in 13 ml of epoxy-hardener at a 2 : 1 ratio. The formed self-healing coating was vacuumed to remove buried air and coated on already cleaned carbon steel coupons, which had been cut to 15 mm × 15 mm × 0.8 mm and polished with sandpapers, washed with distilled water, degreased with ethanol, and sonicated in acetone for 10 min. The coated self-healing sample was tagged CZVEx. Other coating samples were prepared: an epoxy coating containing blank CS microcapsules (CSEx) and a neat epoxy coating (Ex) to serve as the control. The coating was done with a vacuum spin coater (VTC-200, MTI corporation, USA). The coated substrates were left to cure for 72 h in ambient conditions before a cross-scratch was applied with a sharp razor blade making a 10 mm × 10 mm length cross-scratch on the coating down to the substrate and immediately immersed in 3.5 wt% NaCl solution for 14 days.

### 2.6 Electrochemical tests

EIS tests were performed on the cross-scratched samples using a conventional three-electrode system, consisting of the coated metal substrate as the working electrode, an Ag/AgCl electrode as the reference electrode, and an auxiliary platinum electrode as the counter electrode. The analysis was carried out on all the samples at 1, 7, and 14 days of immersion to determine their self-healing and anticorrosion performances. The electrochemical behavior of samples immersed in 3.5 wt% NaCl solution open to air at room temperature was evaluated with electrochemical impedance spectroscopy (EIS) for 14 days of immersion. The test was performed using Gamry Reference 3000 potentiostat at open circuit potential (OCP) after 1 h stabilization. The EIS results were obtained at an amplitude of 10 (mV) over a frequency range of 10 mHz to 100 kHz. The exposed surface area of the coating was 0.785 cm^2^. The Tafel plots of the potentiodynamic polarization were analyzed with Gamry Echem analyst.

## 3 Results and discussion

### 3.1 Characteristics of prepared zirconium vanadate-based material

**Fig. 3:**
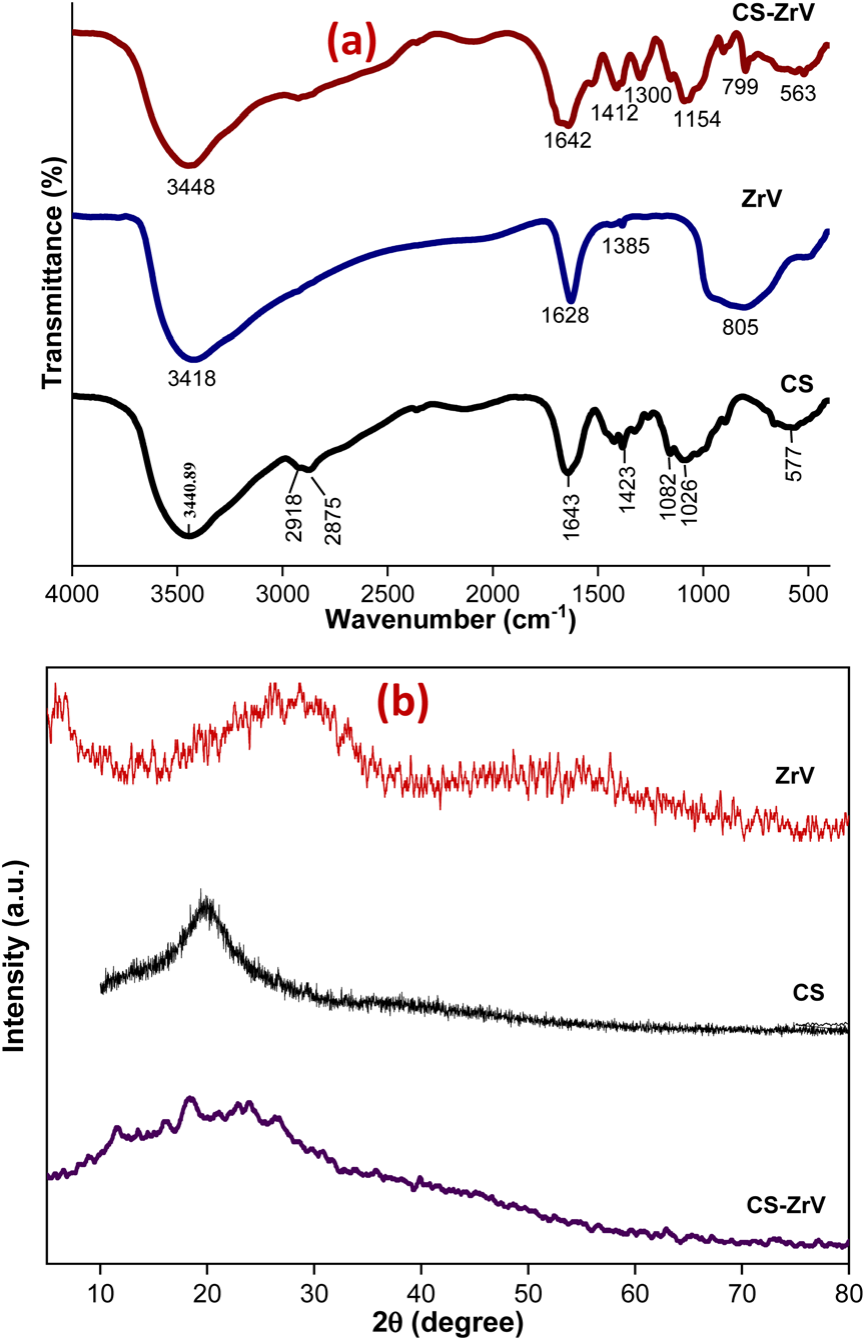
(a) FTIR and (b) XRD spectra of ZrV and CS–ZrV.

The nature of synthesized ZrV and the hybrid microcapsules was studied by FTIR analysis. The ZrV spectrum in Fig. 3(a) confirms the presence of H_2_O, –OH, vanadate ion, and Zr–O–H. Absorption bands of metal oxides are mainly found below 1000 cm^−1^ resulting from inter-atomic vibrations.^31^ The broad and strong band around 3418 cm^−1^ is due to the presence of hydroxyl groups and interstitial water.^32^ The sharp peak at 1628 cm^−1^ is attributed to the deformation vibration of free water molecules. The peak at 1385 cm^−1^ may be ascribed to water bonded with ZrO_2_.^29^ The broad peak at 805 cm^−1^ corresponds to the metal–oxygen vibrational modes bond confirming the formation of ZrV. The spectrum of CS shows the presence of main bands at 3400 cm^−1^ consisting of –OH and –NH_2_ stretching vibrations, at 2918 cm^−1^ corresponding to –CH stretching vibration in –CH and –CH_2_, at 1643 cm^−1^ associated with –NH_2_ bending vibration, at 1423 cm^−1^ due to –CH symmetric bending vibration in –CHOH–, 1082 and 1026 cm^−1^ shows the –CO vibration in –CONH.^33^ The spectrum of CS–ZrV shows that the functional groups of CS were present, and the peak at 1412 cm^−1^ is assigned to the Zr–O stretching frequency; the peak at 799 cm^−1^ may be associated with the presence of V–O vibrations.^34^ The extra peaks at 893 cm^−1^ and 563 cm^−1^ may be associated with the cross-linking action of TPP depicting the PO_4_ groups.

**Fig. 4:**
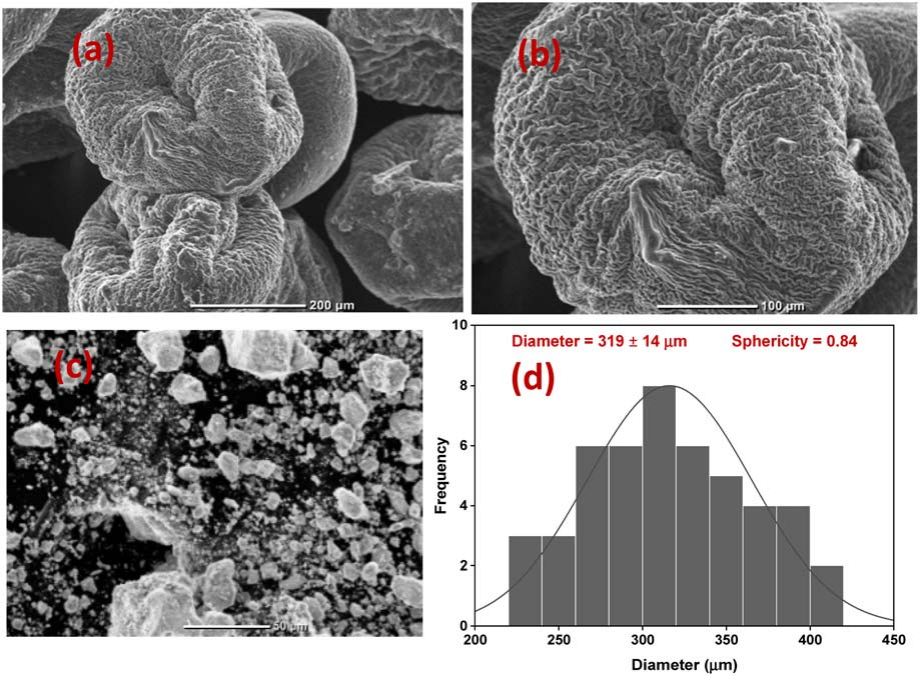
(a and b) Morphology of CS–ZrV microcapsules at different magnifications, (c) SEM micrograph of ZrV nanoparticles (d) size distribution of CS–ZrV microcapsules.

XRD patterns of ZrV nanoparticles and the composite microcapsules are presented in Fig. 3(b). The nanoparticle shows a semi-crystalline structure with two peaks, one at 2*θ* = 9° and the next with a weak diffraction line at 2*θ* of 28.6° indicating minor crystallinity of the compound, which can be described as polycrystalline, assumedly resulting from 1 h heating at 90° during preparation.^20^ The average crystalline diameter was determined to be 47 nm which was calculated from Debye Scherrer's equation:^29^1
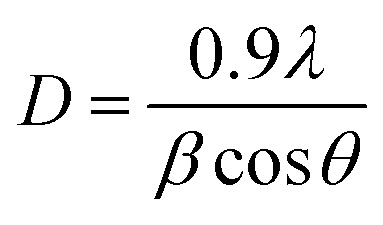
where *D* is the size of the crystallite, *β* is the full-width at half-maximum (FWHM) intensity of the diffraction peak, *θ* is the Bragg's angle and *λ* is the X-ray wavelength. Also, the XRD spectrum of CS shows a characteristic peak of CS at 2*θ* = 20.0° and the ZrV nanoparticle (SEM micrograph shown in Fig. 4(c)) shows the two peaks, which as depicted by the spectrum of CS–ZrV, the positions and intensity of the peaks varied dramatically in contrast to individual CS and ZrV indicating incorporation and interaction of CS with ZrV and the successful crosslinking action of TPP.

The morphology of the composite capsules is shown in Fig. 4(a and b), along with the size distribution curve showing a mean microcapsule size of 319 μm (Fig. 4(d)). The micrograph shows spherical and shrunken morphology with folding patterns, which may be as a result of electrostatic and covalent interaction between CS and ZrV components^35^ along with strong ionic crosslinking action of the TPP.^36^ The surface of the capsules is covered with the ZrV nanoparticles indicating successful immobilization of the particles and formation of composite. The rough and jagged surface of the capsules may provide extra interfacial area and may assist in better adhesion of the coating matrix.^35^ Also, the rough surface aids the microcapsules to have a stronger interface with epoxy coatings thereby reducing debris in wearing conditions.^37^

### 3.2 EDX and thermogravimetric analysis of hybrid microcapsules

**Fig. 5:**
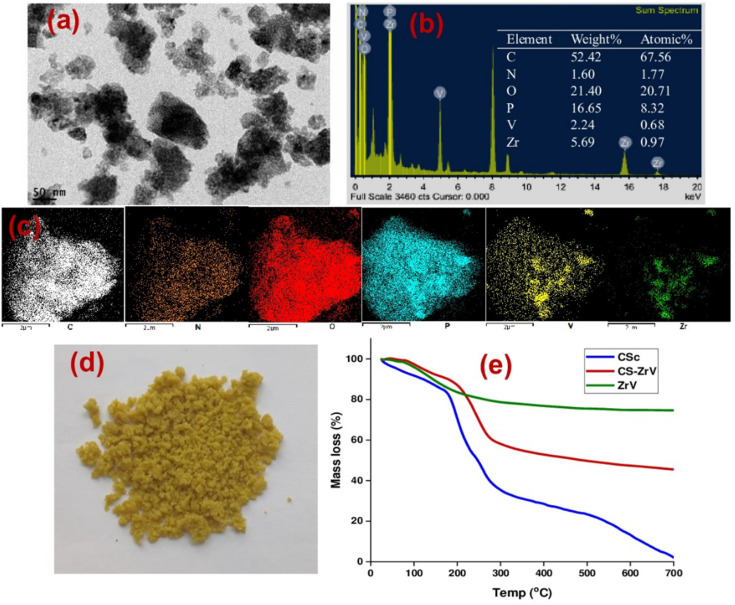
(a) TEM image of ZrV (b) EDX plot and composition of CS–ZrV, (c) mapping of CS–ZrV (d) image of prepared microcapsules, and (e) thermogram of CS–ZrV microcapsules.

TEM-EDX analysis was carried out to determine the nature of the prepared ZrV nanoparticle (Fig. 5(a)) and the elements present in the prepared composite samples are shown in Fig. 5(b). The patterns depict the various elements comprised of the individual materials used to prepare the composite samples. These elements include Zr, O, C, V, and N, along with a significant quantity of phosphate (P) that emanates from the crosslinking action of tripolyphosphate. The mapping (Fig. 5(c)) shows that the CS polymer was sufficiently immobilized with the effective components of Zr and V, confirming the successful preparation and crosslinking of the fabricated composite microcapsules (image shown in Fig. 5(d)).

The TGA analysis is a useful characterization tool that indicates both the thermal stability of modified materials and the successful formation of a new composite. The thermogram of the CS, ZrV, and CS–ZrV in Fig. 5(e) illustrates the thermal degradation of the compounds. The weight loss of around 100 °C depicting an endotherm can be attributed to the loss of adsorbed water and moisture. The weight loss of around 300 °C indicates the thermal degradation of CS.^34^ The curve patterns of ZrV show two main steps of weight loss. The first step represents a weight loss from room temperature to 160 °C due to the removal of free-bond water molecules. The next step thermal loss begins from a temperature above 260 °C and is associated with the removal of interstitial water molecules by condensation of replaceable hydroxyl groups (–OH) in the material, characteristic of synthesized inorganic materials. From 500 °C above, no weight loss was observed indicating that no structural changes occurred in the materials, suggesting that the prepared ZrV is stable up to 700 °C.^29^ The thermogram of the composite microcapsules, CS–ZrV shows a very different thermogram, the initial weight loss at around 100 °C may be attributed to the loss of physically adsorbed water which could not be completely expelled by drying. The first significant weight loss was observed at around 200 °C gradual weight loss at around 500 °C showing the degradation of CS. The remaining mass is attributed to the residual ZrV. Amongst all, the hybrid microcapsules revealed 40% mass loss up to 700 °C, indicating enhanced thermal stability for fabricated composite.

### 3.3 Self-healing and anticorrosion analysis

**Fig. 6:**
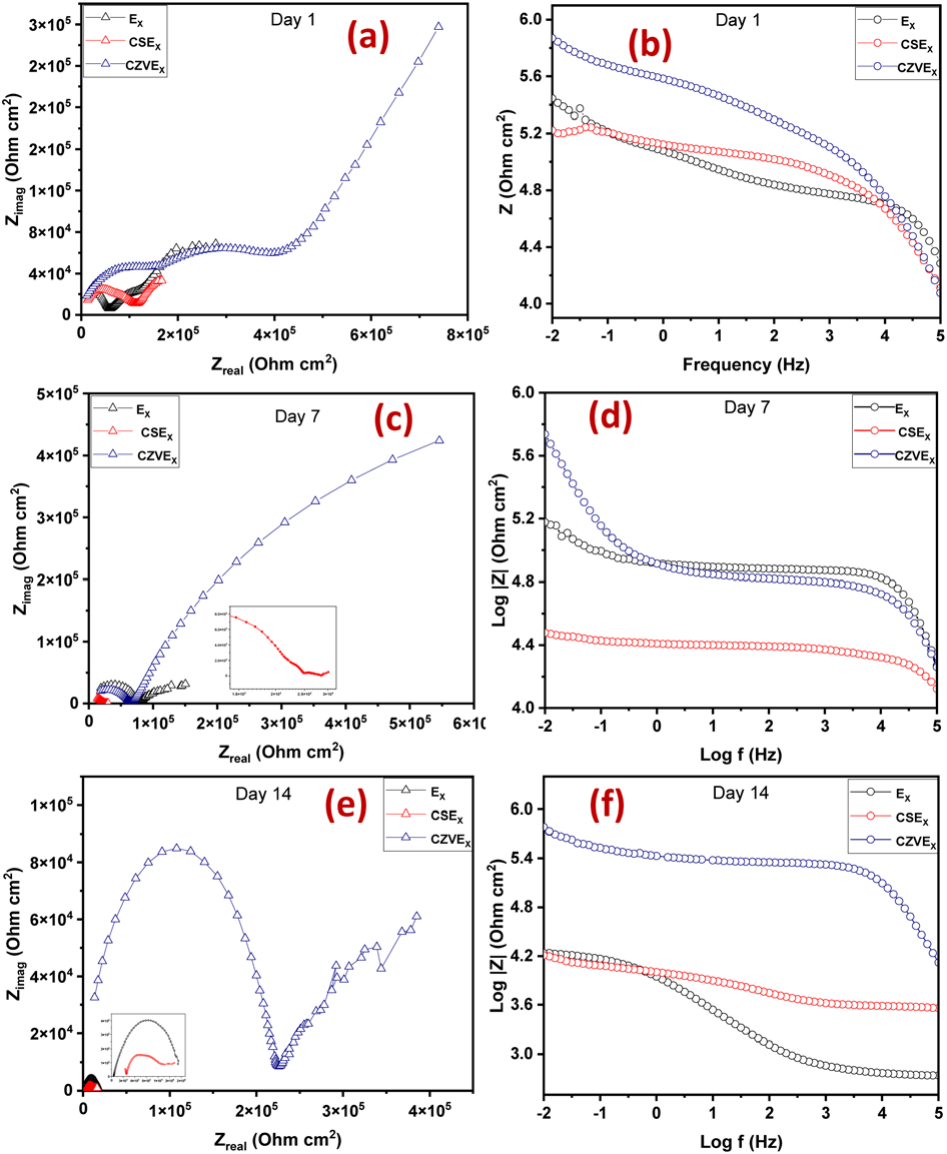
Nyquist and Bode plots of coating samples after (a and b) day 1 (c and d) day 7 and (e and f) day 14 of immersion.

The extent of self-healing and corrosion of the scratched samples subjected to 3.5 wt% NaCl solution for 14 days, with (CZVEx)/without (Ex) the hybrid microcapsules and sample with blank CS microcapsules (CSEx), were measured by EIS to determine the capability of formulated coating to protect carbon steel substrate against corrosion and the self-healing performance. EIS analyses give details on the kinetics of corrosion activity and coating degradation when immersed in corrosive solution. Impedance in the low-frequency range (|Z|_10 mHz_) is an essential parameter for comparing the performances of coatings in corrosion protection studies. A high impedance suggests superior protection.^38^ Thus, EIS allows for evaluation of the level of protection provided by the developed coatings, including information on autonomous healing. Comparing impedance differences across varying immersion times in a corrosive medium can provide preliminary information about self-healing and anticorrosion effects. Resistance of the coatings at different immersion times is depicted by Nyquist and the Bode plot shown in Fig. 6.

The Nyquist plots are observed to have capacitive loops with Warburg impedance extending to the low-frequency regions. Warburg impedance is associated with resistance to mass transfer caused by the formation of an oxide layer on the metal surface. Being that impedance at low frequency suggests corrosion phenomena occurring due to coating deterioration, Nyquist curves with the largest diameter reveal better corrosion resistance. Therefore, as shown in Fig. 6(a and b), after day 1 of immersion, the self-healing coating (CZVEx) showed the highest impedance compared to the other samples. On the other hand, Ex showed higher impedance than CSEx, indicating the introduction of blank CS microcapsules may have increased the porosity of the epoxy coating. Inorganic nanoparticles are regarded as effective corrosion inhibitors.^16^ Thus, the presence of the hybrid microcapsules embedded with the ZrV nanoparticles may have imparted inhibitive properties to the coating as depicted by the left side of the Nyquist curves that associates with the dielectric behavior of the coating and the right side, indicating the charge transfer resistance, equivalent to corrosion resistance.^39^

On the 7th day of immersion, there was a significant decrease in the resistance of the control coatings (Fig. 6(c and d)), while the self-healing coating showed little difference, indicating that its protection ability was quite superior. The little difference in resistance observed may be an indication that the scratched CZVEx has undergone an important reaction relative to the days of immersion. The reaction may have led to the deposition of oxide materials on the scratched interface between electrolyte and substrate, leading to the inception of autonomous healing. Pancrecious *et al.*^40^ noted that the interaction of chloride ions with released vanadate species in a corrosive environment forms polymeric vanadate, which actively impedes the corrosion of metal. It would be observed in the Bode plot (Fig. 6(d)) that there was an instant resistance drop between the frequency range of 10^1^ to 10^4^, which may suggest that although oxide deposits may have impeded the interaction of corrosive ions with the substrate preventing significant corrosion activities on the substrate, autonomous healing of scratched polymer backbone is yet to advance. All the same, CZVEx maintained superior corrosion characteristics and remained at thefifth order of impedance magnitude while the resistance of Ex significantly decreased still maintaining the fifth order and CSEx dropped to the fourth order. However, after the 14th day of immersion (Fig. 6(e and f)), Ex depicted a drastic drop in resistance indicating serious corrosion activities occurring on the substrate due to corrosive agents attack on the scratched coating, which has no self-healing properties. On the other hand, the CSEx sample slightly decreased in resistance, suggesting that the presence of the blank microparticles may have prevented further deterioration of the coating by absorbing corrosive ions and moisture, thereby reducing the diffusional pathway of the coating. Contrastingly, the CZVEx sample (Fig. 6(f)) showed a slight increase in charge transfer resistance compared to that observed on the 7th day of immersion moving from 4.48 × 10^5^ to 5.52 × 10^5^ (ohm cm^2^), suggesting the occurrence of autonomous healing. This could further be observed by the increased resistance around the 10^1^ to 10^4^ frequency regions confirming the healing of the scratched polymer backbone.

**Fig. 7:**
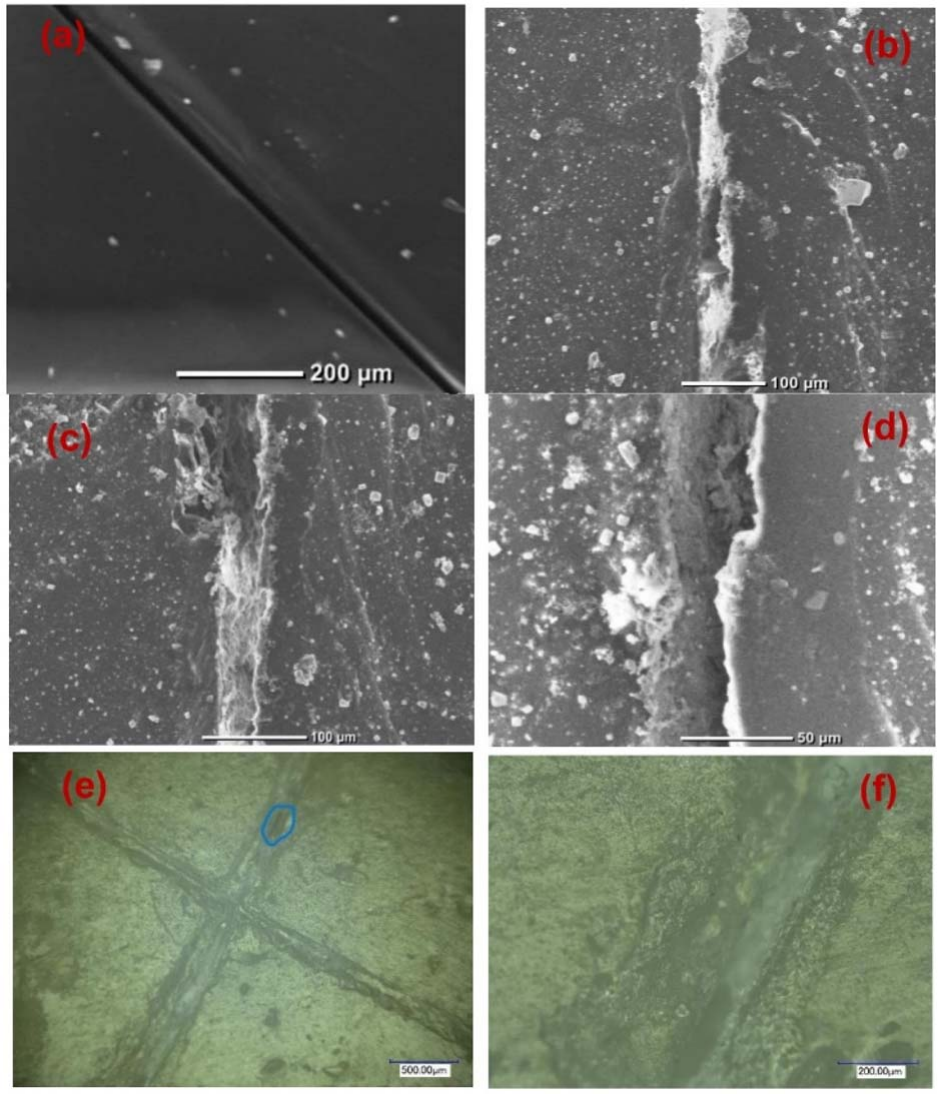
Scratched self-healing coating (a) before immersion, (b–d) after the 14th day of immersion at different magnifications, and (e and f) optical microscope image 1 month after immersion.

SEM images of the scratched CZVEx coating are shown in Fig. 7. The image of the coating before immersion is shown in Fig. 7(a). After 14 days of immersion, the evidence of healing is depicted by the SEM images showing healed scratches spotting tiny regions yet to be healed (Fig. 7(b–d)). The optical microscope images (Fig. 7(e and f)) taken after one month of removing the sample from immersion and left under ambient conditions showed epoxide gel-like substances covering the defects, further confirming the self-healing of the scratched coating. Overall, superior corrosion resistance was observed for the self-healing coating, confirming the effectiveness of the hybrid microparticles in improving the anticorrosion abilities of epoxy coating and the self-healing property due to the synergistic interaction between polymer and nanoparticles, and the formation of polymeric vanadate.

### 3.4 Comparative study on scratched and no-defect CZVEx coating

**Fig. 8:**
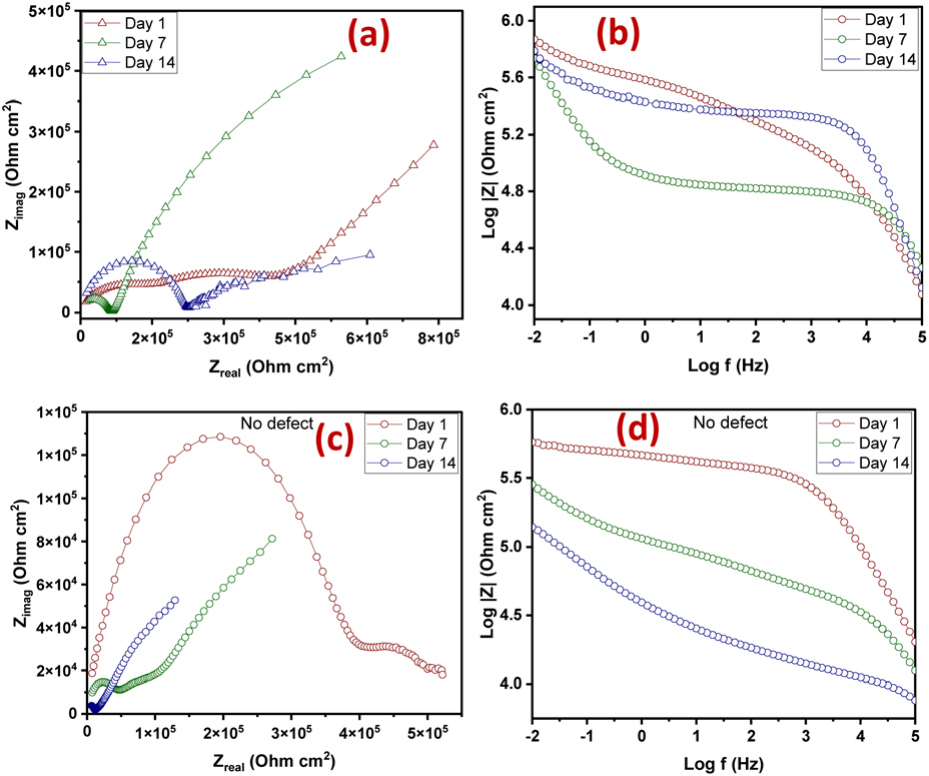
Nyquist and Bode plots of (a and b) scratched (c and d) no-defect coating samples.

The CZVEx coating was studied in a scratched form and without defects. The scratched and no-defect coating samples were analyzed with EIS at 14 days of immersion in a corrosive medium. The essence of inscribing the cross-scratch on the coating is to simulate a coated substrate with microcracks existing within a corrosive environment and to study the probable healing of microcracks in the event of an attack by corrosion agents. Summarily, the scratched sample helps to observe the self-healing property of the coating, while coating without defect helps to monitor the barrier property of the coating. According to Fig. 8(a and b), the Nyquist and Bode curves show the impedance of the scratched coating sample in the fifth impedance order of magnitude for all 1, 7, and 14 days of immersion, with little difference in impedance at the low-frequency regions that is associated with corrosion resistance. This occurrence may be due to the culmination of healing and anticorrosion reactions taking place in the scratched region and coat-substrate interface, verifying the effectiveness of incorporated hybrid microparticles for self-healing and anticorrosion processes.

Notable differences could be observed at the 10^1^ to 10^4^ frequency regions associated with the coating. Within the 1st to 7th day of immersion, the impedance dropped, which of course may be due to the existing defect and implying that no significant repairing action has occurred. However, a significant change was observed after the 14th immersion day where the impedance around the said region increased along with a noticeable increase in resistance at the low-frequency region, indicating both self-healing and improved corrosion resistance. Some studies reported that self-healing abilities found in composite coatings containing vanadate were mainly due to its presence in the coatings and its release through hydrolysis condensation polymerization and the migration of vanadium compounds.^38,41^

On the other hand, the coating sample with no defect (Fig. 8(c and d)) showed a continuous downward impedance spiral across all immersion days, although impedance was maintained at a fifth order of magnitude with a significant difference relative to immersion time. This observation validates the fact that EIS analysis can sufficiently verify the self-healing ability of a coating sample. Also, the reduction in the resistance to corrosion trend observed verifies that the barrier property of a coating and resistance to corrosion is better studied using coatings bearing no defects. The results revealed that the self-healing coating possessed good barrier properties being that it maintained impedance in the fifth order of magnitude even after 14 days of immersion in the corrosive solution. Comparing both scratched samples and samples with no defect, it could be concluded that the CZVEx coating possessed good self-healing and corrosion resistance capabilities. However, studies on conversion coatings formulated with vanadium compound indicated that it possesses more self-healing abilities than barrier protection.^42,43^

**Fig. 9:**
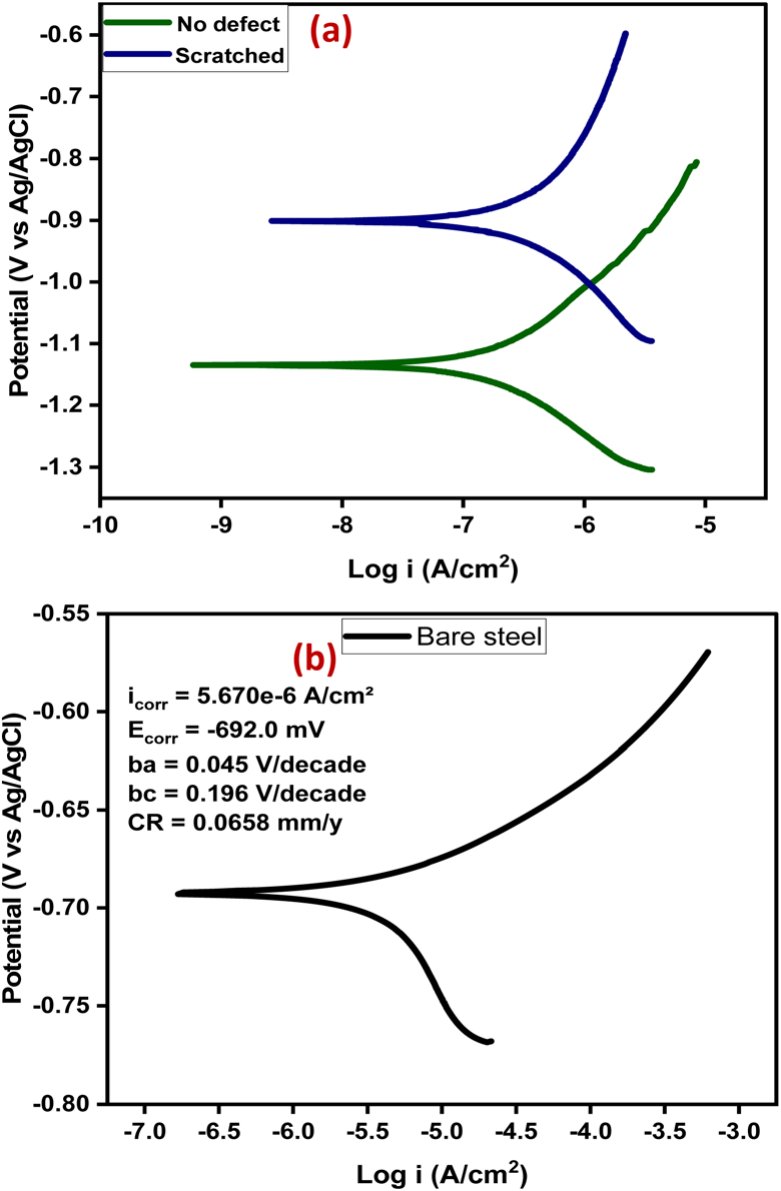
(a) Tafel plots of the samples after 14 days of immersion, (b) Tafel plot of bare steel after 1 day of immersion.

Furthermore, potentiodynamic polarization was carried out, after 14 days of immersion, to study the anti-corrosion performance of the self-healing coating (CZVEx) with/without defect. The tests were carried out in a potential range of ±0.25 V to obtain the Tafel parameters including corrosion current density (*I*_corr_), corrosion potential (*E*_corr_), anodic/cathodic slopes (*b*_a_/*b*_c_), and corrosion rate shown in Table 1. The Tafel plot (Fig. 9(a)) depicts the electrochemical corrosion attributes of the CZVEx coating. Generally, High *E*_corr_ and low *I*_corr_ indicate a better anti-corrosion performance. However, looking at the curves, it is observed that the coating with defect had both higher *E*_corr_ and *I*_corr_ suggesting that it had better anticorrosion performance based on the *E*_corr_, and lower corrosion protection based on *I*_corr_ compared to its counterpart. Similar occurrences could be observed in Tafel plots within some studies.^11,42^

**Table 1:** Tafel parameters of self-healing coatings with/without defects

Parameter	Scratched	No. defect
*I*_corr_ (mA cm^−2^)	6.79 × 10^−7^	2.59 × 10^−7^
*E*_corr_ (mV)	−901	−1140
*B*_a_ (V per decade)	0.575	0.206
*B*_c_ (V per decade)	0.263	0.186
CR (mm per year)	0.007882	0.003005

Researchers^44^ have identified *I*_corr_ as the best kinetic indicator of the corrosion process showing the actual rate of corrosion, *i.e.* it is directly proportional to corrosion rate. On the other hand, *E*_corr_ is a thermodynamic parameter that shows the tendency for corrosion to occur and not the corrosion rate. Taking dressing from the curve of bare steel (Fig. 9(b)) tested after 1 day of immersion, the above statement is confirmed by the *E*_corr_ value that appeared higher than that of the coating samples, and the value that was higher indicating corrosion rate as expected. From the results, the sample with no defect shows better corrosion protection. This is expected, being that there has been a prior corrosion ion attack on the scratched substrate through the defect before healing took place. Overall, both coating samples show good anticorrosion effects at low corrosion rates with the no-defect coating only 2.6 times better than the scratched, indicating successful autonomous healing of the scratched coating. Also, the bare steel shows a strong anodic corrosion reaction, while the curves of coating samples show noticeable impedance to anodic corrosion. This is validated by the Tafel constants (Table 1), which implies the effectiveness of the coating system in controlling anodic and cathodic corrosion reactions when at high values.^3^ The no-defect coating sample shows a high anodic Tafel constant followed by the scratched coating sample, and when compared with the anodic Tafel constant of the bare steel, confirms all previous observations. Overall, the self-healing coating incorporated with CS–ZrV microcapsules possessed good self-healing and anticorrosion capabilities.

### 3.5 Proposed self-healing mechanism of the CZVEx coating

**Fig. 10:**
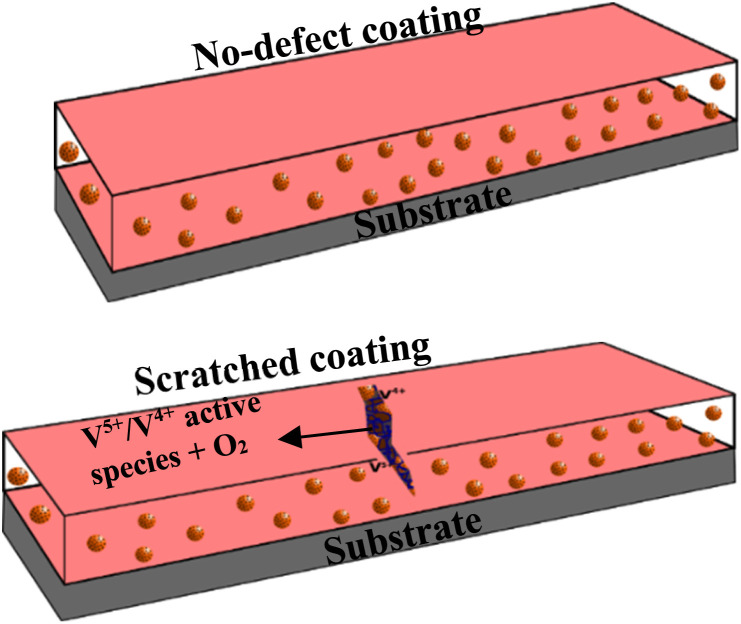
Schematic of the proposed self-healing mechanism of artificially scratched epoxy coating embedding CS–ZrV microcapsules.

Self-healing effects in composite coatings developed with vanadium compound as a component have been attributed to occur due to the migration and release of vanadium compounds, mainly triggered by contact with water and chloride.^41^ The proposed self-healing mechanism of the CZVEx coating is shown in Fig. 10. When the scratched coating is immersed in the corrosive medium, the exposed CS–ZrV microcapsules within the coating around this region interact with corrosive ions and water, which get absorbed by the ZrV-immobilized chitosan microcapsules, triggering the leaching of vanadate from the capsules, and its diffusion to the scratched area. Vanadate is easily leached by water.^45^ The leached vanadate ions react with water and the soluble VO^2+^ forming VO(OH)_3_ by hydrolysis:VO^2+^ + 2H_2_O → VO(OH)_3_ + H^+^

Further reactions of VO(OH)_3_ with water change it to hydrate:VO(OH)_3_ + 2H_2_O → VO(OH)_3_(H_2_O)_2_

The hydrate then polymerizes by forming V^5+^–V^5+^ linkages called polymer backbone. Similarly, vanadate binds through oxygen ligands to form V^5+^–V^4+^ linkages by the same hydrolysis condensation polymerization typical of vanadium conversion coating.^46^ The V^5+^ and V^4+^ active species present at the scratched region may then interact with a corrosive agent, forming a vanadium–peroxo (V^5+^–peroxo) species and V^4+^–OO* radicals, and further reaction with oxygen leads to the formation of epoxide.^47,48^ The optical microscope image in Fig. 7(e and f) provides evidence of epoxide gel-like substances covering the defects. This indicates the self-healing of the scratched coating due to the leaching of the V^5+^–V^5+^ polymer backbone.

## 4 Conclusions

Hybrid chitosan–nanozirconium vanadate (CS–ZrV) microcapsules fabricated using electrospraying techniques, were incorporated into epoxy coating to develop a self-healing coating, CZVEx. Electrochemical analysis of the scratched self-healing coating *via* electrochemical impedance spectroscopy (EIS) showed CZVEx coating had excellent self-healing properties when the impedance of scratched coating increased between the 7th day and 14th day of immersion indicating autonomous repair of the damaged coating from leaching of vanadate and subsequent formation of polymerizable vanadate. Furthermore, potential dynamic polarization analysis of the self-healing coating with and without defect revealed good anticorrosion performance of coatings, with the no-defect coating only 2.6 times more resistant than the scratched coating, as against bare steel that was 22 times more corrosive than the coating with no defect. Hence, the incorporation of the CS–ZrV microcapsules into epoxy coatings induced excellent self-healing anticorrosion performance of the coating.

## Data availability

Data for this article, including description of nanoparticle and biohybrid preparation, images and fitted electrochemical data are available at figshare at https://doi.org/10.6084/m9.figshare.25944088.

## Author contributions

LU: methodology, formal analysis and investigation, writing – original draft preparation, writing – review and editing. ME: conceptualization, validation and writing – review and editing.

## Conflicts of interest

There are no conflicts to declare.

## Supplementary Material
